# Supplementary material for: HIV Superinfection Drives De Novo Antibody Responses and Not Neutralization Breadth
Source: Cell Host Microbe. 2018 Oct 10;24(4):593–599.e3. doi: 10.1016/j.chom.2018.09.001 (PMC6185870; doi:10.1016/j.chom.2018.09.001)
Supplement: Document S1. Figures S1–S3 [file mmc1.pdf]

Cell Host & Microbe, Volume *24*

Supplemental Information

HIV Superinfection Drives *De Novo* Antibody Responses and Not Neutralization Breadth

Daniel J. Sheward, Jinny Marais, Valerie Bekker, Ben Murrell, Kemal Eren, Jinal N. Bhiman, Molati Nonyane, Nigel Garrett, Zenda L. Woodman, Quarraisha Abdool Karim, Salim S. Abdool Karim, Lynn Morris, Penny L. Moore, and Carolyn Williamson

**PID**	**Timing of superinfection** (weeks post infection)	**VL at 52 weeks** (copies/ml)	**CD4 count at superinfection** (cells/µl)	**PI-SI env distance**
CAP237	9 (6-11)	11900	522	11.92%
CAP256	13 (11-14)	178000	555	12.53%
CAP281	42 (40-44)	<400	1199	13.99%
CAP334	(42-47)	14900	(458-543)	nd
CAP377	33 (30-37)	337496	462	15.18%

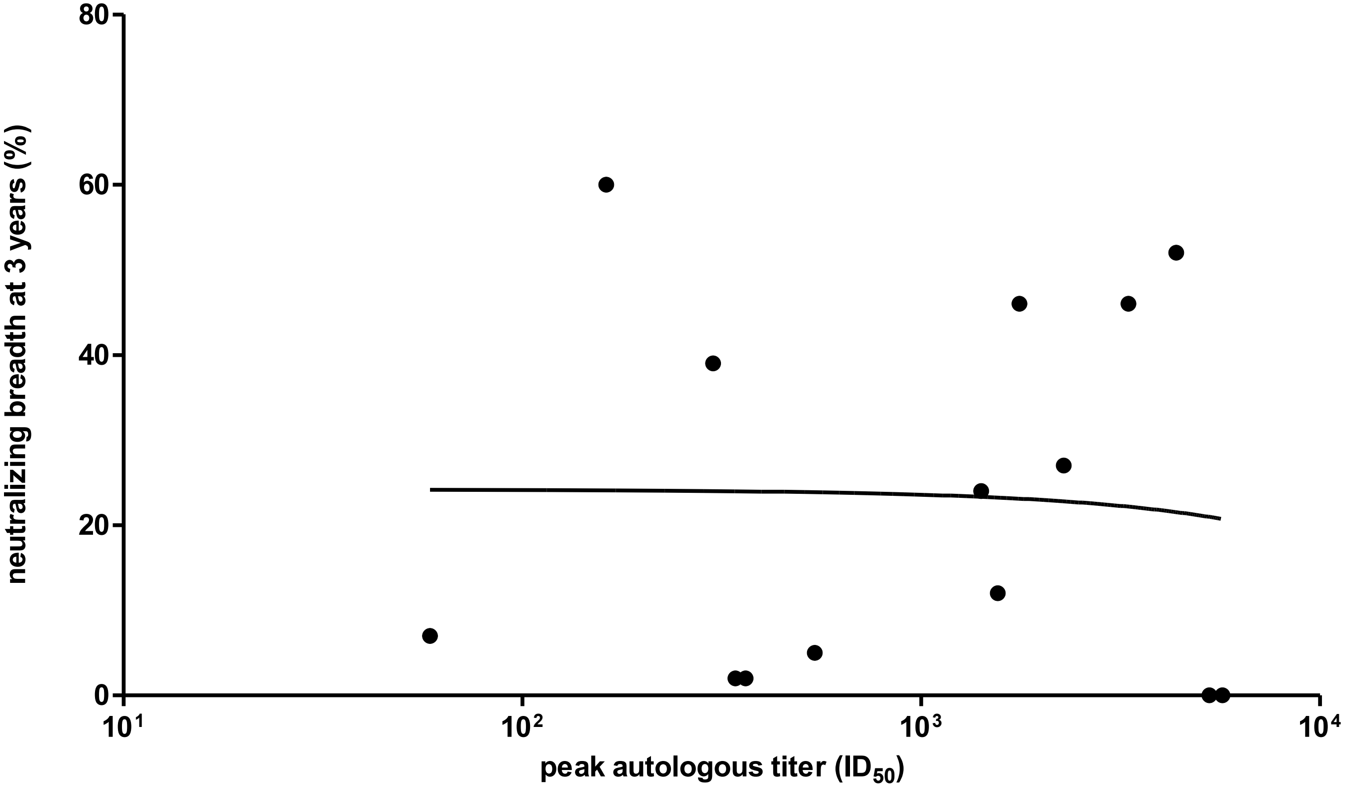

neutralizing breadth at 3 years (%)
80
60
40
20
0
10^1
10^2
10^3
10^4
peak autologous titer (ID_{50})

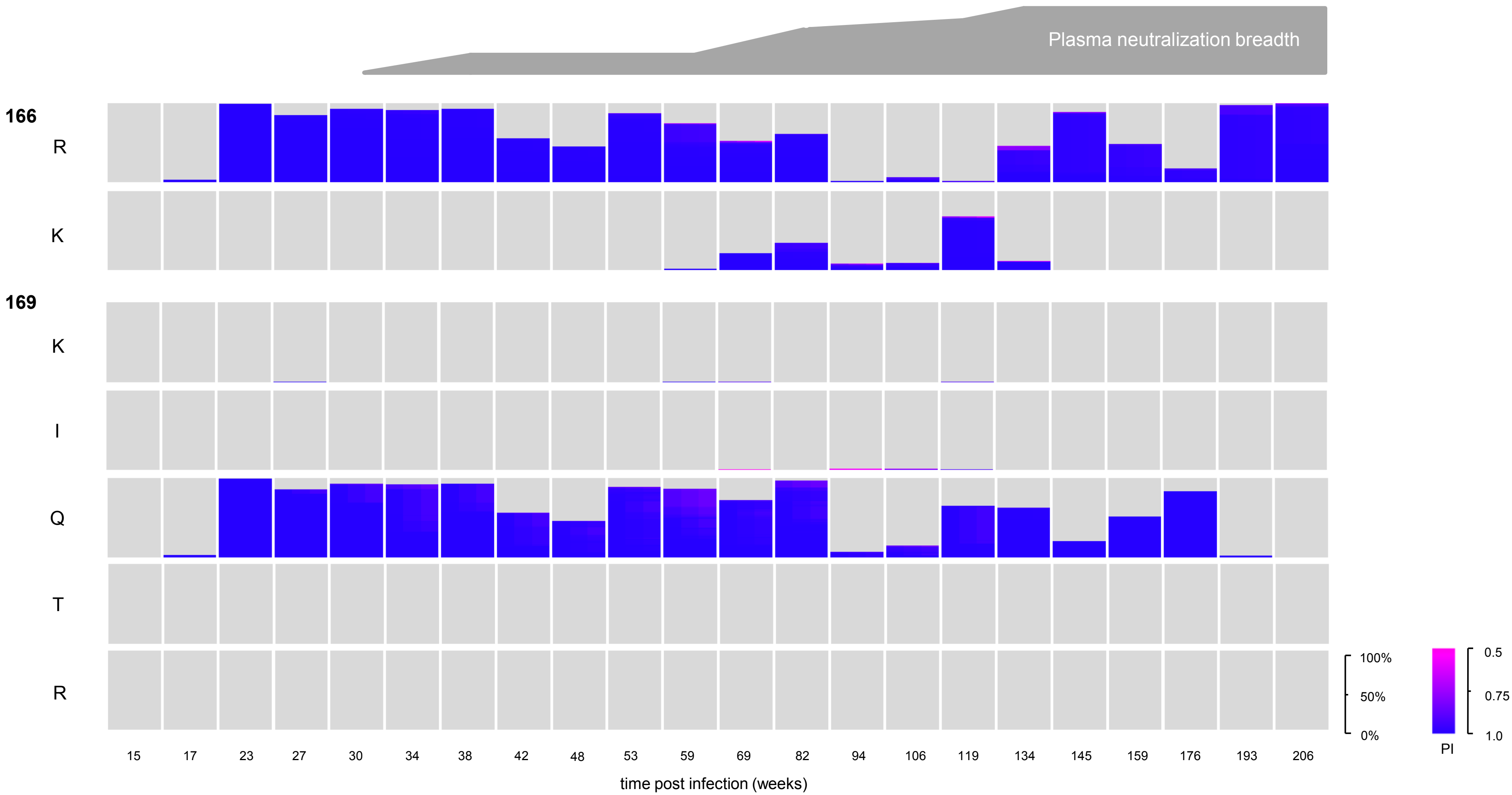

Plasma neutralization breadth
166
R
K
169
K
I
Q
T
R
15
17
23
27
30
34
38
42
48
53
59
69
82
94
106
119
134
145
159
176
193
206
time post infection (weeks)
100%
50%
0%
0.5
0.75
1.0
PI

Figure S1. Identification of superinfection in five participants, Related to Figure 1a. The estimated timing of superinfection, with the confidence interval in parenthesis, is summarized together with the viral load (VL) at 1 year post primary infection, CD4 count at the visit closest to the estimated time of superinfection. PI-SI distance; the DNA distance in *env* between the transmitted/founders of primary infection and superinfection for each participant. nd; not done. PID; anonymized participant ID numbers.

Figure S2. Potency does not lead to breadth, Related to Figure 1b. No significant correlation (P = 0.9559, R^2 = 0.0002) between the potency of the autologous neutralizing antibody response and the later development of neutralization breadth in singly infected participants (n=14). Breadth was measured at 3 years post infection and potency was estimated as the mean of the 3 highest titers observed against an acute/early Env clone.

Figure S3. Frequency of key genotypes in the primary infecting lineage over time, Related to Figure 4. Only residues that were assigned to the primary infecting virus lineage with posterior probabilities >0.5 are included, where more confident assignments are bluer.
