# Supplementary material for: HIV Superinfection Drives De Novo Antibody Responses and Not Neutralization Breadth
Source: Cell Host Microbe. 2018 Oct 10;24(4):593–599.e3. doi: 10.1016/j.chom.2018.09.001 (PMC6185870; doi:10.1016/j.chom.2018.09.001)
Supplement: Document S2. Article plus Supplemental Information [file mmc2.pdf]

Short Article

Cell Host & Microbe

HIV Superinfection Drives *De Novo* Antibody Responses and Not Neutralization Breadth

Graphical Abstract

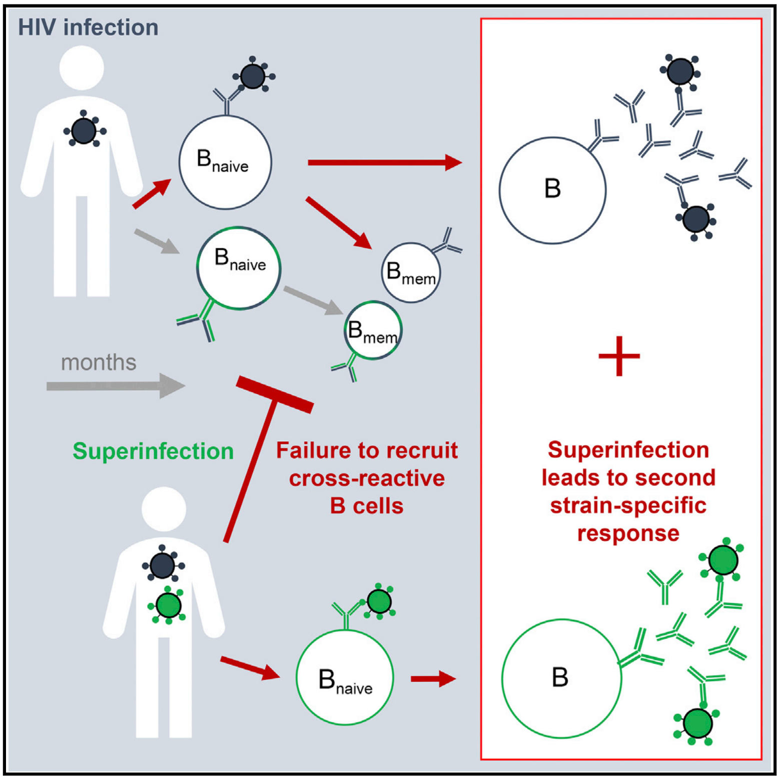

Highlights

- HIV superinfection does not efficiently recruit cross-reactive memory B cells
- Superinfection results in antibody responses specific to each infecting strain
- No evidence that superinfection drives the development of bnAbs

Authors

Daniel J. Sheward, Jinny Marais, Valerie Bekker, ..., Lynn Morris, Penny L. Moore, Carolyn Williamson

Correspondence

pennym@nicd.ac.za (P.L.M.),
carolyn.williamson@uct.ac.za (C.W.)

In Brief

HIV-infected individuals can be re-infected with a second strain (superinfection), providing a model that informs the use of sequential immunizations in future vaccines. Sheward et al. find that superinfection fails to boost memory B cells primed by the first infection and does not promote broadly neutralizing antibodies to HIV.

Sheward et al., 2018, Cell Host & Microbe *24*, 593–599
October 10, 2018 © 2018 The Authors. Published by Elsevier Inc.
https://doi.org/10.1016/j.chom.2018.09.001

CellPress

Cell Host & Microbe
Short Article

HIV Superinfection Drives *De Novo* Antibody Responses and Not Neutralization Breadth

Daniel J. Sheward,[1] Jinny Marais,[1] Valerie Bekker,[2] Ben Murrell,[3] Kemal Eren,[4] Jinal N. Bhiman,[2,5] Molati Nonyane,[2] Nigel Garrett,[6] Zenda L. Woodman,[7] Quarraisha Abdool Karim,[6,8] Salim S. Abdool Karim,[6,8] Lynn Morris,[2,5,6] Penny L. Moore,[2,5,6,10,*] and Carolyn Williamson[1,6,9,10,11,*]

[1]Division of Medical Virology, Department of Pathology, Institute of Infectious Diseases and Molecular Medicine, University of Cape Town, Cape Town 7925, South Africa
[2]Centre for HIV and STI, National Institute for Communicable Diseases of the National Health Laboratory Service, Sandringham, Johannesburg 2192, South Africa
[3]Department of Medicine, University of California, San Diego, San Diego, CA 92093, USA
[4]Bioinformatics and Systems Biology, University of California, San Diego, San Diego, CA 92093, USA
[5]University of Witwatersrand, Johannesburg 2050, South Africa
[6]Centre for the AIDS Programme of Research in South Africa (CAPRISA), University of KwaZulu- Natal, Durban 4013, South Africa
[7]Department of Molecular and Cell Biology, University of Cape Town, Cape Town 7700, South Africa
[8]Department of Epidemiology, Columbia University, New York, NY 10027, USA
[9]National Health Laboratory Services of South Africa, Cape Town 7925, South Africa
[10]These authors contributed equally
[11]Lead Contact
*Correspondence: pennym@nicd.ac.za (P.L.M.), carolyn.williamson@uct.ac.za (C.W.)
https://doi.org/10.1016/j.chom.2018.09.001

SUMMARY

Eliciting antibodies that neutralize a broad range of circulating HIV strains (broadly neutralizing antibodies [bnAbs]) represents a key priority for vaccine development. HIV superinfection (re-infection with a second strain following an established infection) has been associated with neutralization breadth, and can provide insights into how the immune system responds to sequential exposure to distinct HIV envelope glycoproteins (Env). Characterizing the neutralizing antibody (nAb) responses in four superinfected women revealed that superinfection does not boost memory nAb responses primed by the first infection or promote nAb responses to epitopes conserved in both infecting viruses. While one superinfected individual developed potent bnAbs, superinfection was likely not the driver as the nAb response did not target an epitope conserved in both viruses. Rather, sequential exposure led to nAbs specific to each Env but did not promote bnAb development. Thus, sequential immunization with heterologous Envs may not be sufficient to focus the immune response onto conserved epitopes.

INTRODUCTION

Eliciting broadly neutralizing antibodies (bnAbs) through immunization remains a primary goal for HIV prevention. While antibodies capable of neutralizing primary HIV isolates have recently been elicited in animal models by vaccination with stabilized HIV envelope glycoprotein (Env) trimers, the responses were narrow, typically only neutralizing viruses that matched the immunizing Env(s) (Crooks et al., 2015; Sanders et al., 2015; Saunders et al., 2017). Due to the tremendous diversity of HIV Env proteins, protective vaccines will need to direct neutralizing antibody (nAb) responses onto more conserved regions on the HIV Env in order to achieve broad cross-neutralization. BnAbs do develop in a subset of HIV-infected individuals and are typically attributable to one or a few antibody specificities targeting conserved epitopes on the HIV Env (Walker et al., 2010). Defining virological and immunological events and pathways that focus responses to conserved epitopes could identify mechanisms that vaccines could leverage.

RESULTS

Infection by a second HIV strain after established primary infection (HIV superinfection) has been associated with broader antibody responses (Cortez et al., 2012; Powell et al., 2010). Such events are analogous to heterologous prime-boost immunizations, offering an opportunity to assess how the human immune system responds to sequential exposure to two distinct HIV Env antigens. In 108 women recruited in acute/early infection and screened for superinfection over approximately 2 years (Centre for the AIDS Programme of Research in South Africa [CAPRISA] 002 cohort), we have identified five superinfected participants (Redd et al., 2014; Sheward et al., 2015). All five were superinfected between 3 and 10 months following primary infection (Figure S1), and two developed antibodies capable of neutralizing heterologous viruses at 2 years post-infection (Figure 1A). Although we have previously detailed the development of extremely potent, bnAbs in one of these superinfected participants, CAP256 (Bhiman et al., 2015; Doria-Rose et al., 2014), the contribution of superinfection itself to the development of breadth was not clear.

 © 2018 The Authors. Published by Elsevier Inc.
This is an open access article under the CC BY license (http://creativecommons.org/licenses/by/4.0/).

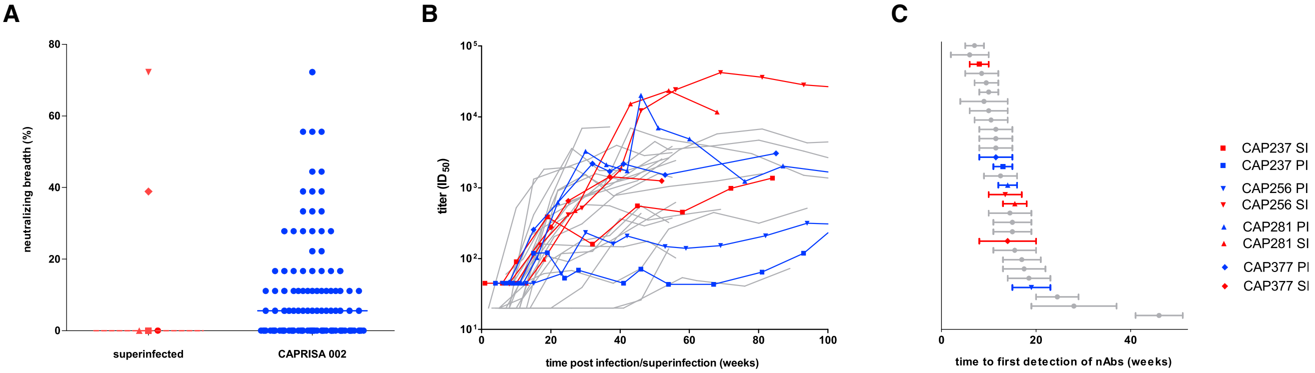

Figure 1. Potent Neutralizing Antibody Responses Arose to Superinfecting Viruses with a Similar Time to Detection as Primary HIV Responses

(A) Comparison of neutralization breadth (percentage of heterologous viruses neutralized) present in plasma sampled 2 years post-infection between superinfected participants (n = 5) and remaining CAP002 cohort participants (n = 119). Antibody breadth was compared at 2 years post-infection as all superinfected participants had at least 2 years of antiretroviral-naive follow-up. Furthermore, if cross-neutralizing antibodies do not develop by 2–3 years post-infection, they are unlikely to do so subsequently (Gray et al., 2011; Landais et al., 2016; Mikell et al., 2011). Cross-neutralization data at 2 years were available for 120 anti-retroviral-therapy-naive participants.

(B) Autologous neutralizing antibody titers, over time, to superinfecting Envs (red), primary infecting Envs in participants later superinfected (blue), and to early/founder Envs from other CAPRISA participants (gray) (n = 22).

(C) Estimated time, in weeks, from transmission until the first detection of these neutralizing antibody responses. The error bars represent the range given the 95% confidence interval for the timing of superinfection.

Studies of nAb responses following superinfection are critically dependent on the identification of the superinfecting Env prior to recombination with the primary infecting virus. Frequent follow-up of participants following diagnosis of infection (every 2 to 4 weeks) enabled us to identify and clone the superinfecting transmitted/founder viruses from four donors (Sheward et al., 2015). The *env* genetic distance between the primary infecting and superinfecting viruses ranged from 11.9% to 15.2%, consistent with unlinked viruses (Figure S1). Despite this intensive sampling, we were unable to obtain the superinfecting variant prior to recombination with the primary virus in the fifth donor (CAP334), highlighting the challenges in accurately deciphering immune responses after superinfection.

Taking advantage of these Env clones, we evaluated whether superinfection promoted responses that cross-neutralized both infecting viruses and whether superinfection may have boosted memory B cell responses primed by an initial infection. Secondary memory responses would be expected to (1) be of higher titer than primary responses, (2) arise more rapidly than primary responses, and (3) neutralize both the priming and boosting antigens (Ahmed and Gray, 1996).

To determine if the nAb responses to superinfection were elevated, we compared the peak nAb titers against all four superinfecting viruses with their matched primary infecting virus, as well as with the nAb titers that increased against early/founder viruses in 22 other participants in the CAPRISA 002 cohort. We found that titers to the CAP237 and CAP377 superinfecting viruses were comparable with those seen in single infections from the rest of the cohort (Figure 1B). In contrast, CAP256 and CAP281 developed exceedingly high neutralizing titers against their superinfecting viruses, with ID_{50} (reciprocal plasma dilution causing 50% reduction of viral infection) titers exceeding 38,000 and 20,000 respectively, that were 24- and 12-fold higher than the cohort median (Figure 1B). In CAP281, these high titers were produced despite maintaining a low or undetectable viral load throughout the first years of infection. However, only CAP256 went on to develop bnAbs, and, among 14 confirmed singly infected participants where neutralization data over 3 years were available, we found no significant association between potency of the autologous nAb response and the later development of breadth (Figure S2). This indicates that eliciting potent responses alone may not be sufficient to promote breadth.

To assess whether the onset of nAb responses to superinfection was accelerated, as would be expected for memory responses, we compared the time from primary infection and superinfection until the first detection of neutralizing antibodies. The nAb responses to superinfecting viruses reached detectable levels a median of 9–17.5 weeks following superinfection (Figure 1C), comparable with the time to first detection of nAbs to the primary infecting viruses in each of the four superinfected individuals. This was also comparable with the timing of nAb responses following primary HIV infection in 22 other participants, which were detectable by a median of 9–16 weeks post-infection (Figure 1C). As the onset of nAbs to the superinfecting viruses were not accelerated, this suggested that they likely represented *de novo* responses, rather than secondary, memory responses.

To determine whether the nAb responses that arose following superinfection were specific for epitopes present in both infecting viruses, we analyzed the dynamics of the nAb responses to primary and superinfecting viruses (Figures 2A–2D). In two donors (CAP281 and CAP237), superinfection was associated with a spike in titers to the primary infecting virus. For both donors, there was also a simultaneous boost in V3 antibodies as measured by ELISA (Figures 2E and 2F) and a transient increase in viral load at this time (Figures 2G and 2H), suggesting that the spikes in titer may have been the result of a generalized activation. However, while titers to the primary viruses increased

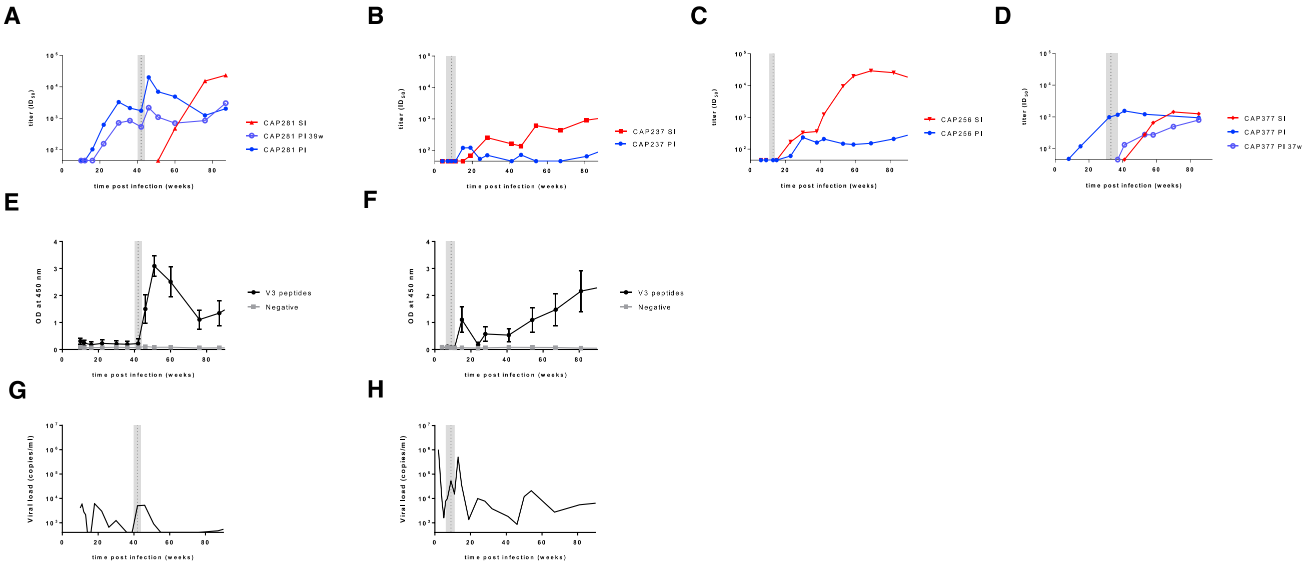

Figure 2. Neutralizing Antibody Responses to the Superinfecting Envs Did Not Cross-Neutralize the Primary Infecting Envs

(A–D) Neutralizing antibody titers, over time, to the primary infecting (PI, blue) and superinfecting (SI, red) Envs from (A) CAP281, (B) CAP237, (C) CAP256, and (D) CAP377. Titers to Env clones representative of the primary infecting variant circulating near the time of superinfection (CAP281 PI 39w in A and CAP377 PI 37w in D), are shown in light blue and with open symbols.

(E and F) Boost in antibodies specific for V3 peptides following superinfection in donors (E) CAP281 and (F) CAP237. Longitudinal V3 antibody titers were determined by ELISA, using six independent clade C V3 peptides. A peptide found in Ebola virus was used as a negative control. Plotted is the mean and SD.

(G and H) Spike in viral load following superinfection. Longitudinal viral load (in copies per milliliter) is depicted for (G) CAP237 and (H) CAP281. The estimated time of superinfection is shown in each figure by the vertical dotted line with the confidence interval shaded.

following superinfection in these donors, they did not cross-neutralize the superinfecting viruses, with nAb titers to the superinfecting viruses only emerging weeks later. Furthermore, when titers to the superinfecting viruses increased, titers to the primary infecting viruses fell (Figures 2A and 2B). Thus, the dynamics of neutralization in both CAP281 and CAP237 are inconsistent with recruitment of memory responses that cross-neutralized both infecting viruses.

In contrast to CAP281 and CAP237, in donors CAP256 and CAP377 nAb titers to the superinfecting viruses and contemporaneous primary viruses increased together (Figures 2C and 2D). This overlapping neutralization profile is suggestive of a response that cross-neutralized both viruses. Furthermore, CAP377 and CAP256 were also the only superinfected individuals that developed neutralization breadth, raising the possibility that superinfection drove breadth by promoting responses to epitopes conserved in both infecting viruses. We therefore mapped the targets of the nAb response following superinfection. As cross-neutralizing activity that developed later in CAP377 was attributable to V2-directed antibodies (Figure 3A), we evaluated whether neutralization of both the primary and superinfecting viruses also targeted V2. The introduction of a 169E mutation into the CAP377 superinfecting virus abrogated neutralization by CAP377 plasma (Figure 3B), indicating that the superinfecting virus was neutralized by V2-directed antibodies dependent on the amino acid at position 169. However, the same mutation in the primary infecting virus had no effect (Figure 3C), indicating that neutralization of the primary virus was mediated by a different neutralizing response to that targeting the superinfecting virus (and mediating breadth). Indeed, the high divergence between the V2s (Figure 3D) provides further evidence that the nAb response did not target an epitope conserved in both viruses. Similarly, the isolation of monoclonal antibodies (mAbs) from CAP256 previously revealed that the precursor of the CAP256-VRC26 antibody lineage (which accounts for the breadth in this donor) neutralized the superinfecting virus but not the primary infecting virus (Doria-Rose et al., 2014). Taken together, these data indicate that the nAb responses to superinfection did not represent the boosting of responses primed by initial infection and did not target epitopes present in the primary infecting viruses.

As superinfection in CAP256 did not drive breadth by promoting multiple responses (breadth was attributable to a single antibody lineage), or by promoting a response to an epitope conserved in both infecting viruses, we investigated whether diversity introduced through superinfection, via recombination, may have facilitated the development of bnAbs in CAP256. We showed previously that acquiring the ability to tolerate diversity at V2 residues 166 and 169, specifically 166K, and 169I/T/Q, was associated with the development of breadth in CAP256 (Bhiman et al., 2015; Doria-Rose et al., 2014). We therefore applied a Hidden Markov Model (STAR Methods) to identify whether this diversity was inherited from the primary infecting virus via recombination or arose independently. We show that 169T and 169I predominantly arose in the superinfecting V2 (Figure 4), and, while 166K and 169Q were inherited from the primary infecting virus (Figure S3), these mutations also arose independently in the superinfecting lineage (Figure 4). These data indicate that multiple infection was not necessary to introduce the diversity that promoted the development of bnAbs in CAP256.

A

	CAP45	CAP45 K169E	CAP45 N160A
CAP377 (2 year plasma)	4527	36	1947

B C

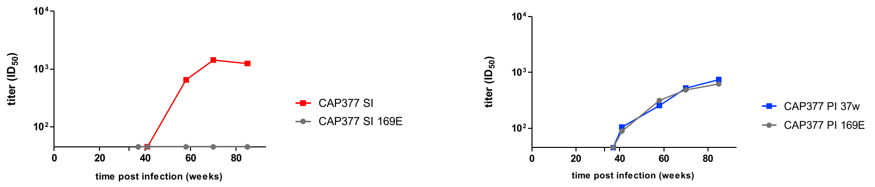

D

```
     157         169                          196
PI   CSFNATTEIKDRKRKVNALFYKLDIVP--------------INETLKIYRLINC
SI   ....T....R.K.QQQY.....P.L..LKNENNSRITVVANSSSNYTD.I....
```

Figure 3. A Single Antibody Response Does Not Account for Neutralization of Both the Primary Infecting and Superinfecting Viruses in Donor CAP377

(A) Introduction of a K169E mutation abrogated neutralization of CAP45 by CAP377, indicating that heterologous neutralization was attributable to a K169-dependent antibody response. Tabled are the ID_{50} titers for each Env tested against a 2 year plasma sample from CAP377.

(B and C) Introduction of a K169E mutation knocks out neutralization of the superinfecting virus (B), but not the primary infecting virus (C), by CAP377 plasma.

(D) High divergence between the CAP377 primary infecting and superinfecting V2 sequences. Numbers represent reference HXB2 numbering.

DISCUSSION

Immunization with multiple Env immunogens has been proposed as a strategy to broaden neutralizing responses. Our identification of the viruses that superinfected CAP256 and three additional superinfected participants in the extremely well-characterized CAPRISA 002 cohort has allowed us to define the neutralizing antibody response to HIV superinfection; a model for heterologous prime-boost immunizations. This has also enabled us to systematically investigate mechanisms whereby superinfection could enhance neutralization breadth: by promoting responses to an epitope conserved in both viruses, by stimulating multiple antibody responses, or by accelerating diversification within epitopes.

A broad diversity of memory B cells are generated in HIV infection (Scheid et al., 2009) that could be activated upon re-infection. However, we show that HIV superinfection likely did not boost nAb memory responses primed by the initial infection. While two of the four superinfected donors developed unusually high autologous titers to the superinfecting virus, none of the nAb responses to superinfecting viruses arose more rapidly than primary HIV responses. Rather, *de novo* nAb responses arose following superinfection. Furthermore, superinfection did not elicit responses to epitopes conserved in both infecting viruses. Overall, infection with two variants resulted in additive responses, although in one case there was a pronounced decline in the titers to the primary infecting virus following superinfection. These findings have important implications for HIV immunizations, including the potential of heterologous prime-boost strategies to activate memory B cell populations. It is important here to differentiate such prime-boost immunizations with unrelated Envs (analogous to superinfection) from sequential, lineage-based immunizations. The latter approach seeks to utilize highly related immunogens that recapitulate viral evolution known to shape antibody lineages toward breadth (Bhiman et al., 2015; Bonsignori et al., 2016; Doria-Rose et al., 2014; Landais et al., 2017; Liao et al., 2013; MacLeod et al., 2016).

While one superinfected donor (CAP256) developed extremely potent bnAbs, we find no evidence that superinfection itself promoted their development. Breadth in CAP256 was attributable to a single antibody lineage (Doria-Rose et al., 2014), indicating that superinfection did not promote breadth by expanding the number of distinct responses. Early antibodies in this lineage were also specific for the superinfecting virus and not the primary infecting virus, indicating that superinfection did not facilitate breadth by directing responses to an Env conserved in both infecting viruses. This is also consistent with observations from the recent isolation of mAbs from another superinfected individual that developed bnAbs; mAbs isolated from this individual neutralized either primary viruses or superinfecting viruses, with none cross-neutralizing both viruses (Williams et al., 2018). In this individual, broadly neutralizing activity also appeared to be largely attributable to the mAb lineage that arose to the superinfecting virus, with the antibodies that arose to the primary infecting virus only making a minor contribution. While acquiring the ability to tolerate viral diversity that arose at key residues led to the CAP256.VRC26 antibody lineage developing broadly neutralizing activity, infection with two strains was not necessary to introduce this diversity. As a result, we find no evidence that superinfection itself was necessary for the development of breadth in CAP256.

Previous studies have reported that superinfection broadens the nAb response (Cortez et al., 2012; Powell et al., 2010), though others saw no statistically significant effect (Cornelissen et al., 2016). Where a significant broadening was observed in previous studies, the effect size was small, with superinfected individuals approximately 1.5 times more likely to develop breadth than singly infected individuals (Cortez et al., 2012). This effect size would be consistent with a near-additive effect of superinfection on nAb breadth, such as that we describe here. It is crucial to differentiate an additive effect (i.e., stimulation of two strain-specific antibody responses, each with the potential to develop breadth) from one that is more likely to be synergistic (e.g., the promotion of a cross-neutralizing antibody response to an epitope conserved on both viruses). Stimulating independent antibody responses to each of two immunizing immunogens has restricted value in the context of a goal to elicit bnAbs to HIV by vaccination.

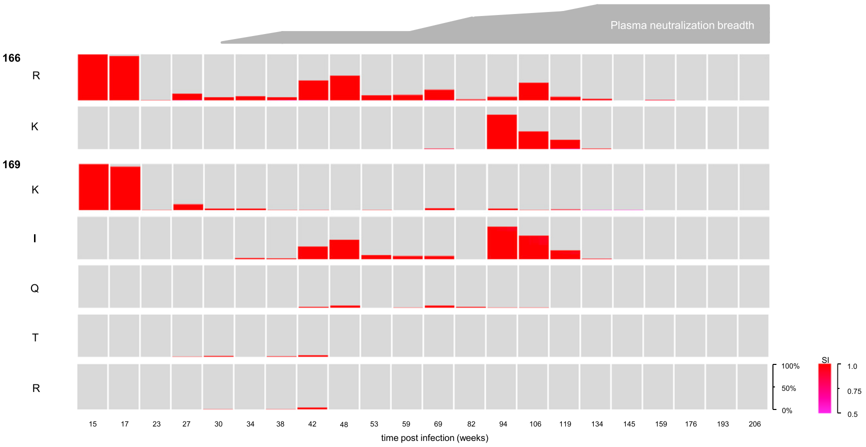

Figure 4. Diversity Associated with Broadening of the Antibody Response in CAP256 Arose in the Superinfecting Virus

Key genotypes and their frequencies in the superinfecting lineage are shown over time. Only residues that were assigned to the superinfecting lineage with posterior probabilities >0.5 are included, where more confident assignments are redder. Overlaid is a schematic of the development of breadth over time.

As HIV superinfection is a rare event, we (and others) have been limited by small sample sizes. Large-scale screening of multiple longitudinal cohorts for cases of superinfection, together with more sophisticated screening approaches, will be required to establish larger sample sizes and could provide extremely valuable insights for sequential immunizations. Furthermore, sequencing of HIV-specific B cells before and after superinfection in the future could identify whether superinfection reactivates any minor memory B cell populations that would not be evident in plasma studies, or whether superinfection leads exclusively to *de novo* responses.

It is also important to consider the on-going HIV replication and immune dysfunction present in HIV-infected individuals that would not be present in the vaccine setting. While memory B cell dysfunction is known to arise with HIV pathogenesis, all participants described here were superinfected within the first year of infection and had relatively high CD4 counts (>450 cells/μL) at the time of superinfection. Host factors, including autoimmunity, may also contribute to the development of broadly neutralizing HIV antibodies (Borrow and Moody, 2017; Dugast et al., 2017; Havenar-Daughton et al., 2016; Landais et al., 2016; Moody et al., 2016), and these were not assessed here. Despite these caveats, results from vaccine studies have thus far been consistent with our observations. Polyvalent and sequential immunization of rabbits with two, three, or four stabilized HIV Env trimers elicited nAbs to each of the Envs in many of the animals, but no significant broadening of the responses to Envs not included in the immunization was evident (Klasse et al., 2016; Torrents de la Peña et al., 2018).

Interestingly, the nAb responses that arose in two individuals to their superinfecting viruses were the most potent responses observed in the cohort. We showed that these were not secondary, memory responses but rather potent, *de novo* responses. Immune complexes are known to be more immunogenic than antigen alone (Brady, 2005), and presentation of superinfecting antigen would have occurred in the context of (non-neutralizing) antibodies produced in response to primary infection. Indeed, in a therapeutic trial, passive therapy with an HIV-specific, broadly neutralizing mAb significantly improved subsequent neutralizing antibody responses in all but one of 15 participants (Schoofs et al., 2016), potentially mediated by a similar mechanism. Identifying the mechanism underlying the potent response to superinfecting viruses could improve future vaccines. In conclusion, we find that HIV superinfection fails to efficiently recruit neutralizing memory B cells and, at best, results in additive nAb responses rather than a synergistic effect leading to cross-neutralization; a distinction that is highly relevant for vaccine design. This suggests that, while sequential immunizations with heterologous Env immunogens may be able to improve the potency of elicited responses, alone, they are unlikely to promote the development of bnAbs.

STAR★METHODS

Detailed methods are provided in the online version of this paper and include the following:

- KEY RESOURCES TABLE
- CONTACT FOR REAGENT AND RESOURCE SHARING
- EXPERIMENTAL MODEL AND SUBJECT DETAILS
  - Human Subjects
  - Cell Lines
- METHOD DETAILS
  - Single Genome Sequencing
  - Molecular Cloning
  - Neutralization Assays
  - V3 ELISA
  - Mutagenesis
  - Recombination Inference in CAP256
- QUANTIFICATION AND STATISTICAL ANALYSIS
- DATA AND SOFTWARE AVAILABILITY
  - Code Availability
  - Data Availability

SUPPLEMENTAL INFORMATION

Supplemental Information includes three figures and can be found with this article online at https://doi.org/10.1016/j.chom.2018.09.001.

ACKNOWLEDGMENTS

We thank the participants in the CAPRISA 002 and 004 cohort for their commitment to attending the CAPRISA clinics in South Africa. Thanks to clinical and laboratory staff at CAPRISA for managing the cohort and providing specimens. We acknowledge research funding from the Center for the AIDS Program of Research (CAPRISA) (S.S.A.K. and Q.A.K.) and the South African Medical Research Council (MRC) SHIP program (P.L.M. and C.W.). CAPRISA is funded by the South African HIV/AIDS Research and Innovation Platform of the South African Department of Science and Technology, and was initially supported by

the US National Institute of Allergy and Infectious Diseases, NIH, US Department of Health and Human Services grant U19 AI51794 (S.S.A.K.). This research was conducted as part of the DST-NRF Center of Excellence in HIV Prevention, which is supported by the DST and the NRF (COE141028106922). This study was supported in part by the Bill & Melinda Gates Foundation's Collaboration for AIDS Vaccine Discovery (CAVD)/ Comprehensive Antibody Vaccine Immune Monitoring Consortium (OPP ID: 1032144 and OPP ID: 1146996). D.J.S. acknowledges support from a CFAR pilot grant and the CAVD Science Exchange Program, and received funding assistance from the Poliomyelitis Research Foundation and the National Research Foundation. B.M. was supported by R00AI120851 from the National Institute of Allergy and Infectious Diseases of the NIH. P.L.M. is supported by the South African Research Chairs Initiative of the Department of Science and Technology and the NRF (grant no. 98341).

AUTHOR CONTRIBUTIONS

D.J.S., P.L.M., and C.W. designed the study, analyzed the data, and wrote the manuscript. D.J.S. and J.M. performed single-genome sequencing. D.J.S., V.B., J.N.B., and M.N. performed neutralization assays. B.M. and K.E. performed the recombination inference. J.N.B. performed the ELISAs. L.M. and Z.L.W. contributed to study design. L.M. contributed to data analysis. N.G. managed the CAPRISA cohort and contributed clinical samples and data. S.S.A.K. and Q.A.K. established and led the CAPRISA cohorts and contributed samples. All authors reviewed and edited the manuscript.

DECLARATION OF INTERESTS

The authors declare no competing interests.

Received: May 7, 2018
Revised: July 10, 2018
Accepted: September 4, 2018
Published: September 27, 2018

REFERENCES

Abdool Karim, Q., Abdool Karim, S.S., Frohlich, J.A., Grobler, A.C., Baxter, C., Mansoor, L.E., Kharsany, A.B.M., Sibeko, S., Mlisana, K.P., Omar, Z., et al. (2010). Effectiveness and safety of tenofovir gel, an antiretroviral microbicide, for the prevention of HIV infection in women. Science *329*, 1168–1174.

Abrahams, M.-R., Anderson, J.A., Giorgi, E.E., Seoighe, C., Mlisana, K., Ping, L.-H., Athreya, G.S., Treurnicht, F.K., Keele, B.F., Wood, N., et al. (2009). Quantitating the multiplicity of infection with human immunodeficiency virus type 1 subtype C reveals a non-Poisson distribution of transmitted variants. J. Virol. *83*, 3556–3567.

Ahmed, R., and Gray, D. (1996). Immunological memory and protective immunity: understanding their relation. Science *272*, 54–60.

Bhiman, J.N., Anthony, C., Doria-Rose, N.A., Karimanzira, O., Schramm, C.A., Khoza, T., Kitchin, D., Botha, G., Gorman, J., Garrett, N.J., et al. (2015). Viral variants that initiate and drive maturation of V1V2-directed HIV-1 broadly neutralizing antibodies. Nat. Med. *21*, 1332–1336.

Bonsignori, M., Zhou, T., Sheng, Z., Chen, L., Gao, F., Joyce, M.G., Ozorowski, G., Chuang, G.-Y., Schramm, C.A., Wiehe, K., et al. (2016). Maturation pathway from germline to broad HIV-1 neutralizer of a CD4-mimic antibody. Cell *165*, 449–463.

Borrow, P., and Moody, M.A. (2017). Immunologic characteristics of HIV-infected individuals who make broadly neutralizing antibodies. Immunol. Rev. *275*, 62–78.

Brady, L.J. (2005). Antibody-mediated immunomodulation: a strategy to improve host responses against microbial antigens. Infect. Immun. *73*, 671–678.

Cornelissen, M., Euler, Z., van den Kerkhof, T.L.G.M., van Gils, M.J., Boeser-Nunnink, B.D.M., Kootstra, N.A., Zorgdrager, F., Schuitemaker, H., Prins, J.M., Sanders, R.W., et al. (2016). The neutralizing antibody response in an individual with triple HIV-1 infection remains directed at the first infecting subtype. AIDS Res. Hum. Retroviruses *32*, 1135–1142.

Cortez, V., Odem-Davis, K., McClelland, R.S., Jaoko, W., and Overbaugh, J. (2012). HIV-1 superinfection in women broadens and strengthens the neutralizing antibody response. PLoS Pathog. *8*, e1002611.

Crooks, E.T., Tong, T., Chakrabarti, B., Narayan, K., Georgiev, I.S., Menis, S., Huang, X., Kulp, D., Osawa, K., Muranaka, J., et al. (2015). Vaccine-elicited tier 2 HIV-1 neutralizing antibodies bind to quaternary epitopes involving glycan-deficient patches proximal to the CD4 binding site. PLoS Pathog. *11*, e1004932.

Doria-Rose, N.A., Schramm, C.A., Gorman, J., Moore, P.L., Bhiman, J.N., Dekosky, B.J., Ernandes, M.J., Georgiev, I.S., Kim, H.J., Pancera, M., et al. (2014). Developmental pathway for potent V1V2-directed HIV-neutralizing antibodies. Nature *509*, 55–62.

Dugast, A.-S., Arnold, K., Lofano, G., Moore, S., Hoffner, M., Simek, M., Poignard, P., Seaman, M., Suscovich, T.J., Pereyra, F., et al. (2017). Virus-driven inflammation is associated with the development of bNAbs in spontaneous controllers of HIV. Clin. Infect. Dis. *64*, 1098–1104.

Gray, E.S., Moore, P.L., Choge, I.A., Decker, J.M., Bibollet-Ruche, F., Li, H., Leseka, N., Treurnicht, F., Mlisana, K., Shaw, G.M., et al. (2007). Neutralizing antibody responses in acute human immunodeficiency virus type 1 subtype C infection. J. Virol. *81*, 6187–6196.

Gray, E.S., Madiga, M.C., Hermanus, T., Moore, P.L., Wibmer, C.K., Tumba, N.L., Werner, L., Mlisana, K., Sibeko, S., Williamson, C., et al. (2011). The neutralization breadth of HIV-1 develops incrementally over four years and is associated with CD4+ T cell decline and high viral load during acute infection. J. Virol. *85*, 4828–4840.

Havenar-Daughton, C., Carnathan, D.G., Torrents de la Peña, A., Pauthner, M., Briney, B., Reiss, S.M., Wood, J.S., Kaushik, K., van Gils, M.J., Rosales, S.L., et al. (2016). Direct probing of germinal center responses reveals immunological features and bottlenecks for neutralizing antibody responses to HIV Env trimer. Cell Rep. *17*, 2195–2209.

Keele, B.F., Giorgi, E.E., Salazar-Gonzalez, J.F., Decker, J.M., Pham, K.T., Salazar, M.G., Sun, C., Grayson, T., Wang, S., Li, H., et al. (2008). Identification and characterization of transmitted and early founder virus envelopes in primary HIV-1 infection. Proc. Natl. Acad. Sci. USA *105*, 7552–7557.

Klasse, P.J., LaBranche, C.C., Ketas, T.J., Ozorowski, G., Cupo, A., Pugach, P., Ringe, R.P., Golabek, M., van Gils, M.J., Guttman, M., et al. (2016). Sequential and simultaneous immunization of rabbits with HIV-1 envelope glycoprotein SOSIP.664 trimers from clades A, B and C. PLoS Pathog. *12*, 1–31.

Landais, E., Huang, X., Havenar-Daughton, C., Murrell, B., Price, M.A., Wickramasinghe, L., Ramos, A., Bian, C.B., Simek, M., Allen, S., et al. (2016). Broadly neutralizing antibody responses in a large longitudinal sub-Saharan HIV primary infection cohort. PLoS Pathog. *12*, e1005369.

Landais, E., Murrell, B., Briney, B., Murrell, S., Rantalainen, K., Berndsen, Z.T., Ramos, A., Wickramasinghe, L., Smith, M.L., Eren, K., et al. (2017). HIV envelope glycoform heterogeneity and localized diversity govern the initiation and maturation of a V2 apex broadly neutralizing antibody lineage. Immunity *47*, 990–1003.e9.

Liao, H.-X., Lynch, R., Zhou, T., Gao, F., Alam, S.M., Boyd, S.D., Fire, A.Z., Roskin, K.M., Schramm, C.A., Zhang, Z., et al. (2013). Co-evolution of a broadly neutralizing HIV-1 antibody and founder virus. Nature *496*, 469–476.

van Loggerenberg, F., Mlisana, K., Williamson, C., Auld, S.C., Morris, L., Gray, C.M., Abdool Karim, Q., Grobler, A., Barnabas, N., Iriogbe, I., et al. (2008). Establishing a cohort at high risk of HIV infection in South Africa: challenges and experiences of the CAPRISA 002 acute infection study. PLoS One *3*, e1954.

MacLeod, D.T., Choi, N.M., Briney, B., Garces, F., Ver, L.S., Landais, E., Murrell, B., Wrin, T., Kilembe, W., Liang, C.-H., et al. (2016). Early antibody lineage diversification and independent limb maturation lead to broad HIV-1 neutralization targeting the Env high-mannose patch. Immunity *44*, 1215–1226.

Martin, D.P., Murrell, B., Golden, M., Khoosal, A., and Muhire, B. (2015). RDP4: detection and analysis of recombination patterns in virus genomes. Virus Evol. *1*, vev003.

Mikell, I., Sather, D.N., Kalams, S.A., Altfeld, M., Alter, G., and Stamatatos, L. (2011). Characteristics of the earliest cross-neutralizing antibody response to HIV-1. PLoS Pathog. *7*, e1001251.

Moody, M.A., Pedroza-Pacheco, I., Vandergrift, N.A., Chui, C., Lloyd, K.E., Parks, R., Soderberg, K.A., Ogbe, A.T., Cohen, M.S., Liao, H.-X., et al. (2016). Immune perturbations in HIV-1–infected individuals who make broadly neutralizing antibodies. Sci. Immunol. *1*, aag0851.

Powell, R.L.R., Kinge, T., and Nyambi, P.N. (2010). Infection by discordant strains of HIV-1 markedly enhances the neutralizing antibody response against heterologous virus. J. Virol. *84*, 9415–9426.

Rabiner, L.R. (1989). A tutorial on hidden Markov models and selected applications in speech recognition. Proc. IEEE *77*, 257–286.

Redd, A.D., Mullis, C.E., Wendel, S.K., Sheward, D., Martens, C., Bruno, D., Werner, L., Garrett, N.J., Abdool Karim, Q., Williamson, C., et al. (2014). Limited HIV-1 superinfection in seroconverters from the CAPRISA 004 microbicide trial. J. Clin. Microbiol. *52*, 844–848.

Sanders, R.W., van Gils, M.J., Derking, R., Sok, D., Ketas, T.J., Burger, J.A., Ozorowski, G., Cupo, A., Simonich, C., Goo, L., et al. (2015). HIV-1 neutralizing antibodies induced by native-like envelope trimers. Science *349*, aac4223.

Saunders, K.O., Verkoczy, L.K., Jiang, C., Zhang, J., Parks, R., Chen, H., Housman, M., Bouton-Verville, H., Shen, X., Trama, A.M., et al. (2017). Vaccine induction of heterologous tier 2 HIV-1 neutralizing antibodies in animal models. Cell Rep. *21*, 3681–3690.

Scheid, J.F., Mouquet, H., Feldhahn, N., Seaman, M.S., Velinzon, K., Pietzsch, J., Ott, R.G., Anthony, R.M., Zebroski, H., Hurley, A., et al. (2009). Broad diversity of neutralizing antibodies isolated from memory B cells in HIV-infected individuals. Nature *458*, 636–640.

Schoofs, T., Klein, F., Braunschweig, M., Kreider, E.F., Feldmann, A., Nogueira, L., Oliveira, T., Lorenzi, J.C.C., Parrish, E.H., Learn, G.H., et al. (2016). HIV-1 therapy with monoclonal antibody 3BNC117 elicits host immune responses against HIV-1. Science *352*, 997–1001.

Sheward, D.J., Ntale, R., Garrett, N.J., Woodman, Z.L., Abdool Karim, S.S., and Williamson, C. (2015). HIV-1 superinfection resembles primary infection. J. Infect. Dis. *212*, 904–908.

Torrents de la Peña, A., de Taeye, S.W., Sliepen, K., LaBranche, C.C., Burger, J.A., Schermer, E.E., Montefiori, D.C., Moore, J.P., Klasse, P.J., and Sanders, R.W. (2018). Immunogenicity in rabbits of HIV-1 SOSIP trimers from clades A, B, and C, given individually, sequentially, or in combination. J. Virol. *92*, e01957-17.

Walker, L.M., Simek, M.D., Priddy, F., Gach, J.S., Wagner, D., Zwick, M.B., Phogat, S.K., Poignard, P., and Burton, D.R. (2010). A limited number of antibody specificities mediate broad and potent serum neutralization in selected HIV-1 infected individuals. PLoS Pathog. *6*, e1001028.

Williams, K.L., Wang, B., Arenz, D., Williams, J.A., Dingens, A.S., Cortez, V., Simonich, C.A., Rainwater, S., Lehman, D.A., Lee, K.K., et al. (2018). Superinfection drives HIV neutralizing antibody responses from several B cell lineages that contribute to a polyclonal repertoire. Cell Rep. *23*, 682–691.

STAR★METHODS

KEY RESOURCES TABLE

REAGENT or RESOURCE	SOURCE	IDENTIFIER
Antibodies		
alkaline phosphatase-labeled goat anti-human (Fc-specific) antibody	Sigma-Aldrich	Cat#A0170; RRID: AB_257868
Biological Samples		
Patient-derived plasma samples	van Loggerenberg et al., 2008; Abdool Karim et al., 2010	https://www.caprisa.org/
Critical Commercial Assays		
Viral RNA mini kit	QIAGEN	Cat#52906
Bright-Glo luciferase assay system	Promega	Cat#E2650
Quikchange Lightning mutagenesis kit	Agilent	Cat#210519
Platinum Taq High Fidelity	Invitrogen	Cat#11304029
pcDNA3.1 directional topo cloning kit	Invitrogen	Cat#K4900-40
Experimental Models: Cell Lines		
TZM-bl cell line	NIH-ARP Cat# 8129-442	RRID: CVCL_B478
HEK293T cell line	Laboratory of George Shaw (University of Alabama, Birmingham, AL).	RRID: CVCL_0063
Oligonucleotides		
5'- GGGTTTATTACAGGGACAGCAGAG -3'	Integrated DNA Technologies (IDT)	N/A
5'-GCACTCAAGGCAAGCTTTATTGAGGCTTA -3'	Integrated DNA Technologies (IDT)	N/A
5'- CACC GGCTTAGGCATCTCCTATAGCAGGAAGAA-3'	Integrated DNA Technologies (IDT)	N/A
5'- TTGCCAATCAAGGAAGTAGCCTTGTGT -3'	Integrated DNA Technologies (IDT)	N/A
Recombinant DNA		
pSG3Äenv	https://www.aidsreagent.org/	NIH ARP Cat#11501
Software and Algorithms		
Custom HMM algorithm	Martin et al., 2015 and this paper	https://github.com/MurrellGroup/recombination-hmm

CONTACT FOR REAGENT AND RESOURCE SHARING

Further information and requests for resources and reagents should be directed to and will be fulfilled by the Lead Contact, Carolyn Williamson (carolyn.williamson@uct.ac.za).

EXPERIMENTAL MODEL AND SUBJECT DETAILS

Human Subjects

Samples were provided from participants from the CAPRISA 002 Acute Infection Study established in 2004 (van Loggerenberg et al., 2008). This was an observational cohortthat recruited recently HIV infected women from high risk, HIV negative women from Durban and Vulindlela, KwaZulu-Natal, South Africa (CAP237, CAP256, CAP281) as well as from the CAPRISA 004 study upon seroconversion (CAP334 and CAP377) (Abdool Karim et al., 2010). CAP334 received a 1% Tenofovir gel vaginal microbicide and CAP334 received a placebo vaginal microbicide prior to enrolment as part of the CAPRISA 004 study, that were discontinued upon HIV infection. Ages at enrolment of CAP237, CAP256, CAP281, CAP334 and CAP377 were 25, 19, 53, 25, and 21 years respectively. The timing of infection was estimated as either the midpoint between the last antibody negative and first antibody positive visits, or 14 days prior to an RNA-positive, antibody negative sample. HIV positive participants were followed longitudinally, and plasma samples were taken weekly for three weeks, fortnightly until approximately three months post infection, monthly until approximately 1 year post infection, and quarterly thereafter. Plasma was stored in either EDTA, or ACD (acid citrate dextrose) to prevent coagulation, and stored at -80°C until use. Participants in this study were antiretroviral therapy (ART) naïve and were initiated on ART consistent with the prevailing South African ART guidelines. CD4+ T cell counts at the time of superinfection are shown in Figure S1.

Ethical approval for this study was received from the ethics committees of the University of Cape Town (025/2004), the University of KwaZulu-Natal (E013/04), and the University of the Witwatersrand (MM040202), and all participants provided written, informed consent.

Cell Lines

TZM-bl (JC53-bl) cells (sex: female), engineered by J. Kappes and X. Wu, were obtained from the NIH AIDS Research and Reference Reagent Program (cat# 8129). HEK293T cells (sex: female) were obtained from George Shaw (University of Alabama, Birmingham, AL). Both cell lines were maintained in Dulbecco's Modified Eagle Medium (Gibco, Life Technologies, Carlsbad, CA), containing 4.5 g/L glucose, L-glutamine, sodium pyruvate, and supplemented with 50 μg/ml Gentamicin (Sigma-Aldrich, St Louis, MO), 25 mM HEPES (Sigma-Aldrich, St Louis, MO), and 10% heat inactivated Fetal Bovine Serum (FBS) (Biochrom, Cambridge, UK). Cells were cultured at 37°C in a humidified incubator with 5% CO_2, and monolayers were disrupted at confluence with Trypsin-EDTA.

METHOD DETAILS

Single Genome Sequencing

Plasma viral RNA was extracted from 200 μl or 400 μl of plasma using either the Roche MagNApure (Roche Applied Science, Mannheim, Germany) or QIAamp viral RNA mini kits (Qiagen, Valencia, CA). RNA was reversed-transcribed to cDNA using Superscript III (Invitrogen, Life Technologies, Carlsbad, CA) as per the manufacturer's instructions. *Env* cassettes were amplified from the cDNA in a nested PCR by Single Genome Amplification (SGA) using 0.025 units of Platinum Taq High Fidelity (Invitrogen, Life Technologies, Carlsbad, CA) per 20 μl reaction, as previously described (Abrahams et al., 2009; Keele et al., 2008). Primers used in the outer reaction were 5'- GGGTTTATTACAGGGACAGCAGAG -3' (HXB2 nt 4900 - 4923) and 5'-GCACTCAAGGCAAGCTTTATTGAGGCTTA -3' (HXB2 nt 9604 - 9632). Inner primers used were 5'- CACC GGCTTAGGCATCTCCTATAGCAGGAAGAA-3' (HXB2 nt 5954 - 5982) and 5'- TTGCCAATCAAGGAAGTAGCCTTGTGT -3' (HXB2 nt 9145-9171). Outer reaction thermal cycling conditions were as follows: initial denaturation at 94°C for 2 minutes, followed by 35 cycles of [94°C for 15 seconds, 55°C for 30 seconds, 68°C for 4 minutes], followed by a final extension for 1 cycle at 68°C for 20 minutes. Inner reaction thermal cycling conditions were the same as above, for 45 cycles. Amplicons were directly sequenced using an ABI3000 genetic analyser and BigDye terminator reagents (Applied Biosystems, Foster City, CA) using twelve primers, by the Central Analytic Facility at the University of Stellenbosch, South Africa. Contigs were assembled using Sequencher 4.10.1 (Gene Codes, Ann Arbor, MI). All sequences were screened for contamination against a database of all sequences generated in the laboratory including all constructs used.

Molecular Cloning

Amplicons were ligated into pcDNA3.1-Topo Directional Cloning Vector (Invitrogen, Life Technologies, Carlsbad, CA), and transformed into Top10 Chemically Competent *E. Coli* cells (Invitrogen, Life Technologies, Carlsbad, CA) as per the manufacturer's instructions, and cultured on Luria-Bertani agar supplemented with 100 μg/ml Carbenicillin (Sigma-Aldrich, St Louis, MO). Plasmid purification was performed using the QIAprep Spin Miniprep, or the QIAfilter Plasmid Midi kits (Qiagen, Valencia, CA), as per the manufacturer's instructions. Plasmids were sequenced as described above in order to ensure the clone contained no non-synonymous mutations relative to the SGA-derived *env* sequence.

Neutralization Assays

Env-pseudotyped viruses were generated by co-transfecting *env* plasmids with pSG3ΔEnv at a 1:2 ratio into HEK293T cells using Fugene 6 (Applied Science, Indianapolis, IA) or PolyFect (Qiagen, Valencia, CA) per the manufacturer's instructions. Pseudoviruses were harvested from the supernatant 48 hours following transfection, filtered through a 0.45 μm filter (Millipore, Merck, Billerica, MA), made up to 20% FBS, and stored at -80°C until use. Neutralization assays were performed as described previously (Gray et al., 2007). Assays were performed in duplicate wells, and repeated at least twice using aliquots of the same plasma draw. Neutralization breadth for the cohort was previously characterized, and quantified as the proportion of heterologous viruses from a multi-subtype pseudovirus panel of 18 viruses each plasma sample was able to neutralize at ID_{50} titers >45.

V3 ELISA

Six 33-mer V3 peptides, representative of the V3s from CAPRISA participants CAP88, CAP45, CAP239, CAP63, CAP206 and CAP84 were used to estimate V3 antibody titers. Peptides were coated onto high-binding 96-well enzyme-linked immunosorbent assay plates at a concentration of 2.5 μg/ml in sodium bicarbonate buffer (pH 8.5) overnight at 4°C. Unbound peptide was removed by washing four times with phosphate-buffered saline containing 0.3% Tween 20 (wash solution), and plates were blocked for 1 h at room temperature with 200 μl of phosphate-buffered saline, 0.3% Tween 20, and 5% nonfat milk. Serum samples were diluted 1:500 in block solution, and 100 μl per well was added, followed by incubation at room temperature for 1 h. Plates were washed four times with wash solution before the addition of 100 μl of secondary antibody (horseradish peroxidase-labelled goat anti-human (Fc-specific) antibody (Sigma-Aldrich, St. Louis, MO) diluted 1:1,000 in blocking solution), and incubated for 1 h at 37°C. After four washes with wash solution, bound antibody was detected using tetramethylbenzidine (TMB) substrate and stopped by the addition of 25 μl of 1 M sulfuric acid.

Mutagenesis

Point mutants were generated using the Quikchange Lightning site-directed mutagenesis kit (Agilent Technologies, Santa Clara, CA) as per the manufacturer's instructions.

Recombination Inference in CAP256

A Hidden Markov Model (HMM) was used to assign sites to parent viruses, similar to the "BURT" approach used in RDP4 (Martin et al., 2015). The HMM takes as input two parent sequences (*A* and *B*), and one target sequence, and outputs a probabilistic assignment of each nucleotide site in the target sequence to a parent. To achieve this, a hidden state is associated with each parent. Where the model is in state "A", a site in the target sequence has a high probability of matching parent *A*, and a lower probability of mismatching, and similarly for state "B". This framework naturally accounts for the possibility that a base that originated from one parent could stochastically mutate to now match the other parent. These match and mismatch probability parameters, as well as a parent switching-rate parameter, are fit by approximate maximum likelihood, using Viterbi Training (Rabiner, 1989), and the empirical Bayes posterior assignments of sites to parents is calculated using the Forward-Backward algorithm. This was applied to an alignment of V1V2 sequences from CAP256 (BioProject accession number PRJNA294363) (Bhiman et al., 2015), and the posterior probabilities were visualized in Mathematica 10 (Wolfram Research, Champaign, IL).

QUANTIFICATION AND STATISTICAL ANALYSIS

The mean of the three (contiguous) highest titers were used as estimates of peak titers. The relationship between autologous potency and heterologous breadth was assessed using Pearson's correlation using log10 transformed peak titers, implemented in Prism 5 (Graphpad Software, San Diego, CA). Log10 transformed peak titers and breadth values passed the Kolmogorov-Smirnov test for normality, implemented in Prism 5. "n=" represents number of individuals.

DATA AND SOFTWARE AVAILABILITY

Code Availability

The custom HMM was implemented in Python, and the code is available at https://github.com/MurrellGroup/ with no restrictions to access.

Data Availability

The data that support the findings of this study are available from the Lead Contact upon reasonable request.

Cell Host & Microbe, Volume *24*

Supplemental Information

HIV Superinfection Drives *De Novo* Antibody Responses and Not Neutralization Breadth

Daniel J. Sheward, Jinny Marais, Valerie Bekker, Ben Murrell, Kemal Eren, Jinal N. Bhiman, Molati Nonyane, Nigel Garrett, Zenda L. Woodman, Quarraisha Abdool Karim, Salim S. Abdool Karim, Lynn Morris, Penny L. Moore, and Carolyn Williamson

**PID**	**Timing of superinfection** (weeks post infection)	**VL at 52 weeks** (copies/ml)	**CD4 count at superinfection** (cells/µl)	**PI-SI env distance**
CAP237	9 (6-11)	11900	522	11.92%
CAP256	13 (11-14)	178000	555	12.53%
CAP281	42 (40-44)	<400	1199	13.99%
CAP334	(42-47)	14900	(458-543)	nd
CAP377	33 (30-37)	337496	462	15.18%

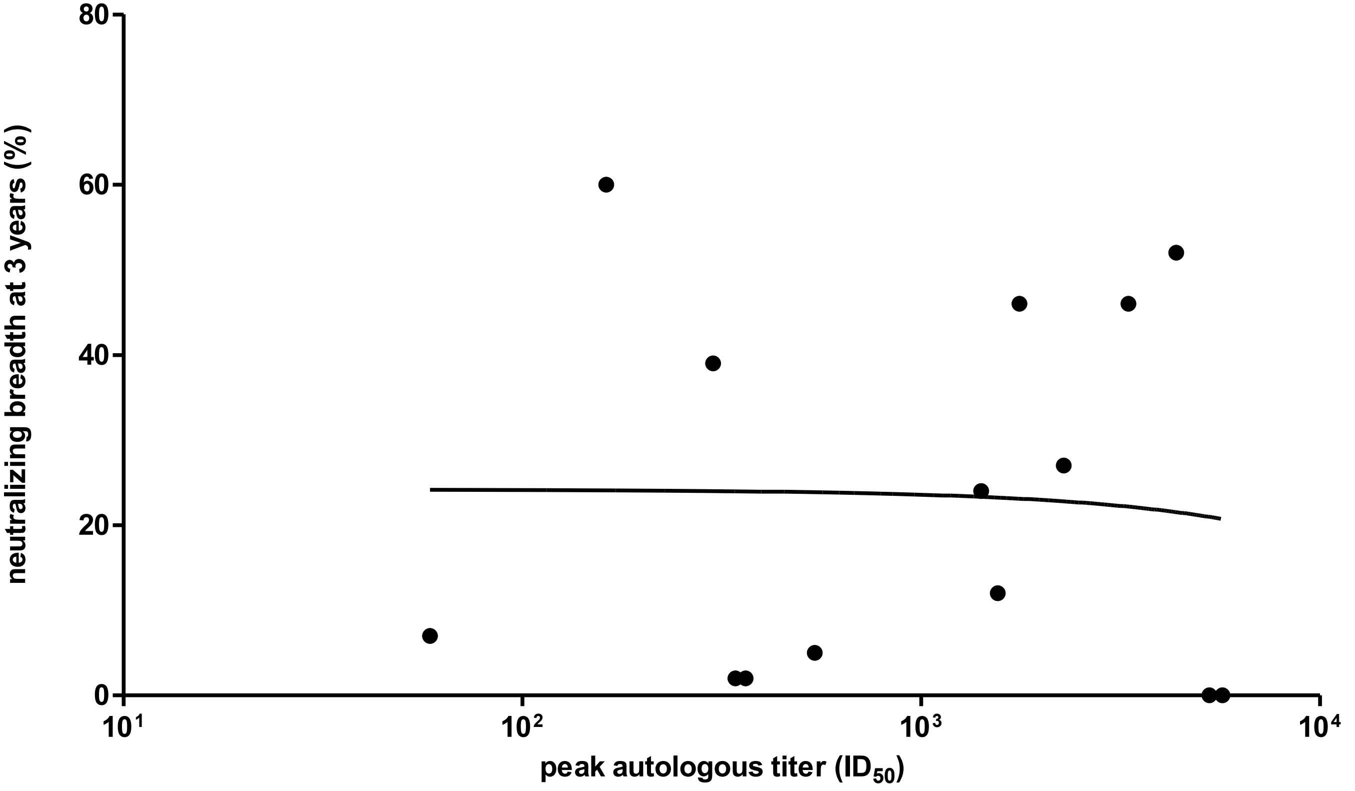

80
60
40
20
0
neutralizing breadth at 3 years (%)
10^1
10^2
10^3
10^4
peak autologous titer (ID50)

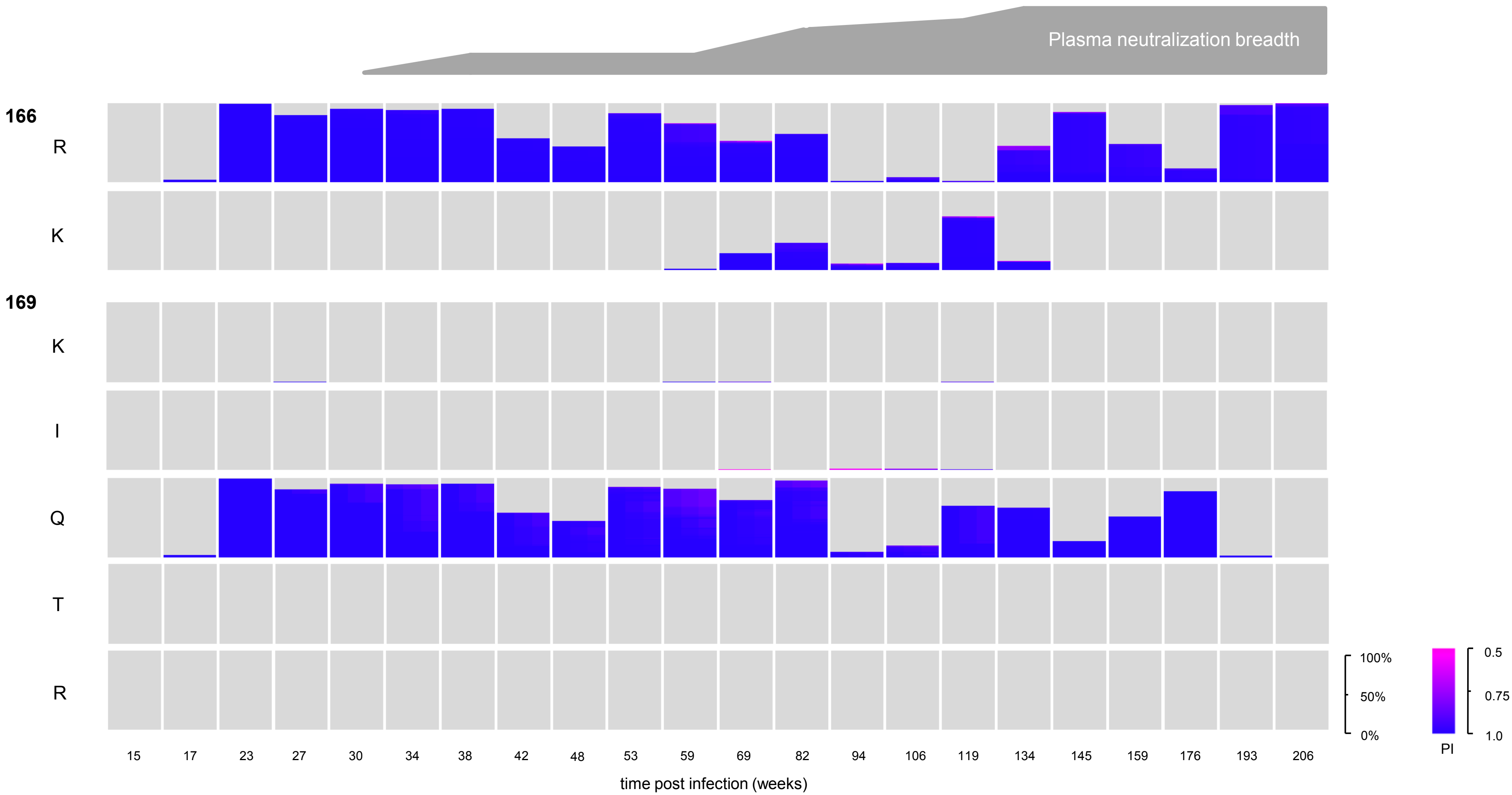

Plasma neutralization breadth
166
R
K
169
K
I
Q
T
R
15
17
23
27
30
34
38
42
48
53
59
69
82
94
106
119
134
145
159
176
193
206
time post infection (weeks)
100%
50%
0%
0.5
0.75
1.0
PI

Figure S1. Identification of superinfection in five participants, Related to Figure 1a. The estimated timing of superinfection, with the confidence interval in parenthesis, is summarized together with the viral load (VL) at 1 year post primary infection, CD4 count at the visit closest to the estimated time of superinfection. PI-SI distance; the DNA distance in *env* between the transmitted/founders of primary infection and superinfection for each participant. nd; not done. PID; anonymized participant ID numbers.

Figure S2. Potency does not lead to breadth, Related to Figure 1b. No significant correlation (P = 0.9559, R^2 = 0.0002) between the potency of the autologous neutralizing antibody response and the later development of neutralization breadth in singly infected participants (n=14). Breadth was measured at 3 years post infection and potency was estimated as the mean of the 3 highest titers observed against an acute/early Env clone.

Figure S3. Frequency of key genotypes in the primary infecting lineage over time, Related to Figure 4. Only residues that were assigned to the primary infecting virus lineage with posterior probabilities >0.5 are included, where more confident assignments are bluer.
